# Proportional sway-based electrotactile feedback improves lateral standing balance

**DOI:** 10.3389/fnins.2024.1249783

**Published:** 2024-03-18

**Authors:** V. S. Raghav Hari Krishna, Jeonghee Kim, Shuo-Hsiu Chang, Yoonsuck Choe, Hangue Park

**Affiliations:** ^1^Department of Computer Science and Engineering, Texas A&M University, College Station, TX, United States; ^2^Department of Electronic Engineering, Department of Biomedical Engineering, and Department of Artificial Intelligence, Hanyang University, Seoul, Republic of Korea; ^3^Department of Physical Medicine and Rehabilitation, University of Texas Health Science Center at Houston, Houston, TX, United States; ^4^Department of Biomedical Engineering, Sungkyunkwan University, Suwon, Republic of Korea; ^5^Department of Intelligent Precision Healthcare Convergence, Sungkyunkwan University, Suwon, Republic of Korea; ^6^Department of Electrical and Computer Engineering, Texas A&M University, College Station, TX, United States

**Keywords:** standing balance, neuromodulation, electrotactile feedback, electrical stimulation, calcaneal nerve stimulation

## Abstract

**Introduction:**

Plantar cutaneous augmentation is a promising approach in balance rehabilitation by enhancing motion-dependent sensory feedback. The effect of plantar cutaneous augmentation on balance has been mainly investigated in its passive form (e.g., textured insole) or on lower-limb amputees. In this study, we tested the effect of plantar cutaneous augmentation on balance in its active form (i.e., electrical stimulation) for individuals with intact limbs.

**Methods:**

Ten healthy subjects participated in the study and were instructed to maintain their balance as long as possible on the balance board, with or without electrotactile feedback evoked on the medial side of the heel, synched with the lateral board sway. Electrotactile feedback was given in two different modes: 1) Discrete-mode E-stim as the stimulation on/off by a predefined threshold of lateral board sway and 2) Proportional-mode E-stim as the stimulation frequency proportional to the amount of lateral board sway. All subjects were distracted from the balancing task by the n-back counting task, to test subjects’ balancing capability with minimal cognitive involvement.

**Results:**

Proportional-mode E-stim, along with the n-back counting task, increased the balance time from 1.86 ± 0.03 s to 1.98 ± 0.04 s (*p* = 0.010). However, discrete-mode E-stim did not change the balance time (*p* = 0.669). Proportional-mode E-stim also increased the time duration per each swayed state (*p* = 0.035) while discrete-mode E-stim did not (*p* = 0.053).

**Discussion:**

These results suggest that proportional-mode E-stim is more effective than discrete-mode E-stim on improving standing balance. It is perhaps because the proportional electrotactile feedback better mimics the natural tactile sensation of foot pressure than its discrete counterpart.

## 1 Introduction

Balance is a well-orchestrated sensorimotor process based on integrated sensory feedback including visual, vestibular, and somatosensory feedback. Therefore, a slight problem in one of those sensory modalities causes a balance issue. For example, compromised sensory feedback from the foot degrades the ability to self-regulate balance which may lead to falling and long-term hospitalization. About 8% of the elderly people, ~60% of the people with diabetes, and ~ 30% of the people who received chemotherapy suffer from peripheral neuropathy and sensory loss at the foot (National Institute of Neurological Disorders and Stroke, 2018).

To address the sensory loss and the ensuing balance problem, multiple motor augmentation approaches have been actively investigated, including exoskeletal assistance or functional electrical stimulation (FES) (Kim et al., 2012; Chen et al., 2013). However, both exoskeleton and FES approaches apply corrective efforts directly to the motor output and bypass the central nervous system (CNS) (Dollahon et al., 2020). The minimal involvement of the CNS may critically limit the neural reorganization necessary for balance enhancement. In another approach, the sensory loss can be indirectly addressed by audiovisual augmentation. Although their efficacy on balance enhancement has been demonstrated, there is still a question on their effect on retention after the therapy ends (Huang et al., 2006; Roemmich et al., 2016). This is perhaps because of the intrinsic heavy cognitive engagement in processing audiovisual feedback, which may raise issues in consistency that are crucial in promoting retention (Andersson et al., 2002; Sigrist et al., 2013). Furthermore, audiovisual feedback is the main sensory modality for communication, and therefore its efficacy can be easily decreased during communication by distraction.

**Figure 1:**
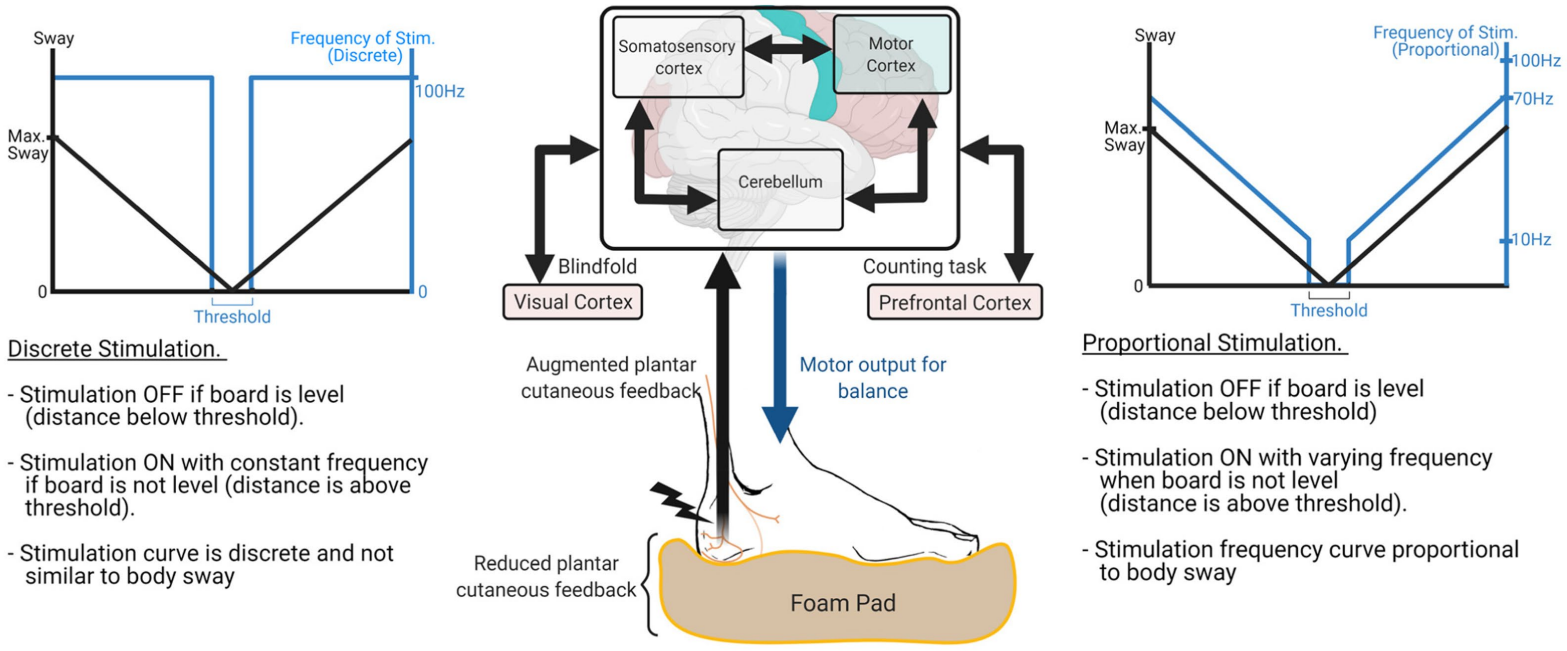
Overall system block diagram to explain the effect of sway-based electrotactile feedback in supplementing proprioceptive feedback under the challenging ground conditions. Signal diagram at the center describes that multiple areas of CNS, including motor cortex, somatosensory cortex, and cerebellum, process plantar cutaneous feedback and generate motor output for balance. Sway-based electrotactile feedback was applied in either discrete or proportional mode. Discrete mode turned on/off stimulation with predefined threshold (left side) and proportional mode changed stimulation frequency according to the body sway (right side).

Peripheral sensory augmentation is a promising approach in balance rehabilitation because it provides motion-dependent sensory feedback, which plays a key role in motor rehabilitation by updating the status of each lower-limb muscle to the CNS in real time (Lynskey et al., 2008; Pearson, 2008; Viseux et al., 2019). Prior works confirmed that motion-dependent sensory feedback is sufficient to engage the plasticity mechanism within the CNS (Takeoka et al., 2014), by reactivating dormant interneurons (Angeli et al., 2018). Also, peripheral sensory augmentation engages the CNS actively in the loop, while motor augmentation engages the CNS passively via the change in motor outcome. The diagram at the center of Figure 1 describes how peripheral sensory augmentation engages the CNS in the loop while motor augmentation does not. Furthermore, for people with peripheral neuropathy, peripheral sensory augmentation on the foot (i.e., plantar cutaneous augmentation) can be a direct solution to addressing the problem (Bernard-Demanze et al., 2009).

Peripheral sensory augmentation has been relatively well investigated for gait rehabilitation after spinal cord injury. For example, body weight-supported treadmill training with robotic exoskeletons repetitively moves paralyzed lower limbs according to a designated walking pattern, in turn generating motion-dependent sensory feedback (Ferris et al., 2005; Hornby et al., 2005). Electrical stimulation applied onto the dura mater augments the somatosensory feedback from the leg and foot, as well as increasing the excitability of interneurons to sensory feedback (Angeli et al., 2018; Wagner et al., 2018). Indeed, there is a strong need to investigate the potential of peripheral sensory augmentation in balance rehabilitation, to utilize it effectively together with the existing techniques (Huang et al., 2006; Sigrist et al., 2013).

Despite the advantages and massive potential, the effect of active plantar cutaneous augmentation on improving balance has been under-investigated for several reasons. First, in many cases, peripheral sensory augmentation has been applied to the body area that is indirectly associated with balance. For example, peripheral sensory augmentation, through wearable devices on the arm or the chest, showed promising results in improving balance (Shull and Damian, 2015; Sorgini et al., 2018; Ballardini et al., 2020). They provide sensory feedback as a sensory cue for the balancing task, and therefore they cannot be free from compromised efficacy in situations with high attentional demands (Azbell et al., 2019). Second, the effect of active plantar cutaneous augmentation on balance has been mainly investigated on lower-limb amputees but not on individuals with intact limbs (Charkhkar et al., 2020; Nanivadekar et al., 2022). Although the results from lower-limb amputees provide important insight, test conditions are different from those of individuals with intact limbs because of the missing sensory feedback from the feet. Third, the effect of plantar cutaneous augmentation on balance has been mainly investigated with passive approaches, such as applying textures or 3D insole under the foot sole (Kelleher et al., 2010; Bae et al., 2016). However, the parameters of passive plantar cutaneous augmentation cannot be actively changed, which limits the degree of freedom in experimental design and customization for each subject.

**Figure 2:**
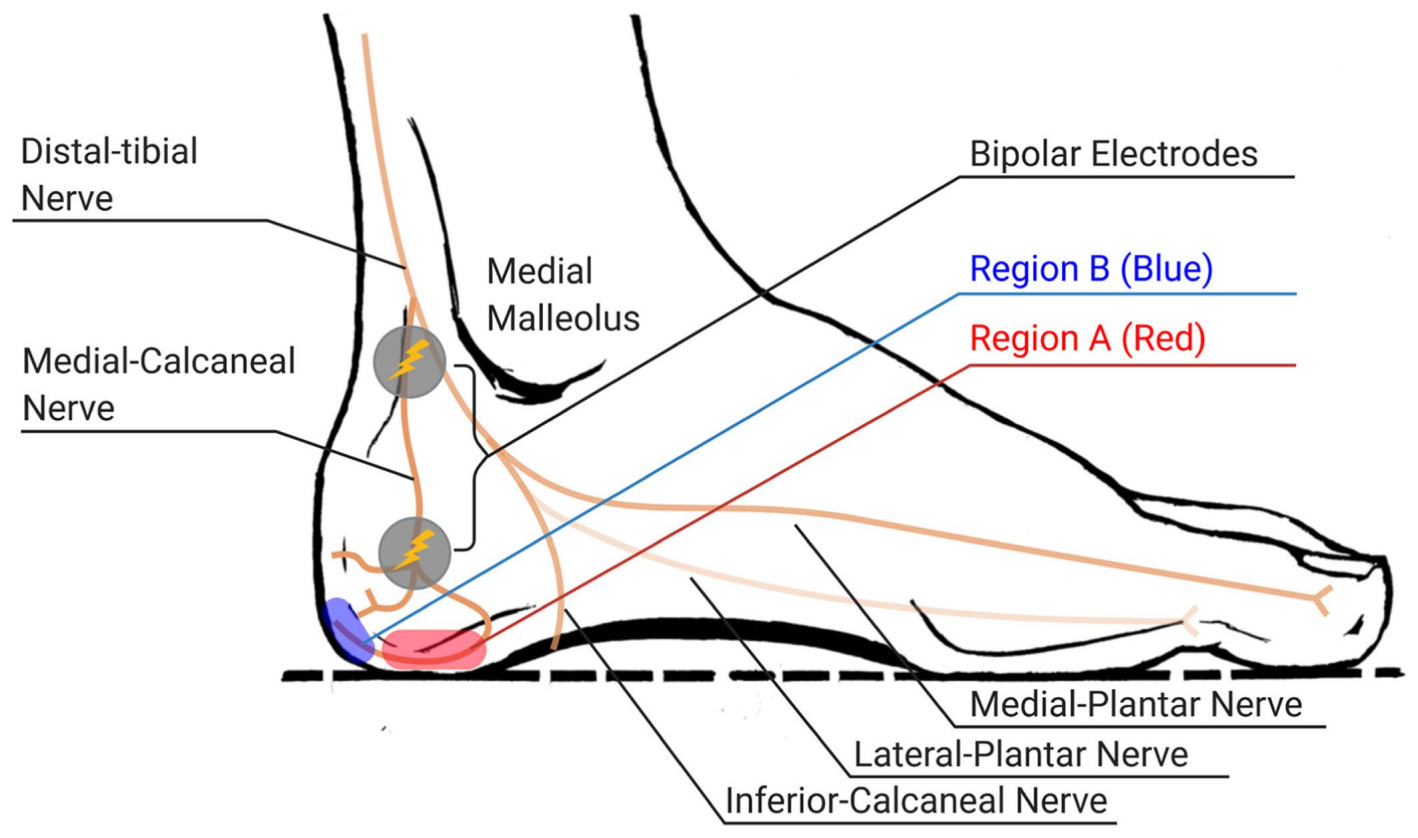
Regions of stimulation over medial-calcaneal nerve and expected locations of evoked electrotactile feedback (regions A and B, painted as red and blue circles respectively, indicate the area around the distal end of each branch of medial-calcaneal nerve).

Electrical stimulation (E-stim) has a great potential to actively augment plantar cutaneous feedback with a high level of controllability, by opening voltage-gated ion channels and generating action potential at sensory axons. Najafi et al. (2017) applied E-stim directly to the foot sole and showed its efficacy in balancing function. However, locating electrodes under the foot sole may disturb the sensitive area of the foot sole, which would not be a good idea as an everyday solution. Indeed, our prior work showed that E-stim applied posterior and/or inferior to the medial malleolus (see Figure 2) can also augment tactile feedback on the foot sole (Kouzaki and Masani, 2008). Therefore, E-stim can augment plantar cutaneous feedback from the foot without disturbing the area of the foot sole. Further, E-stim can be also easily implemented into a small wearable device or even implantable device (Shon et al., 2021) and used in daily lives as well as in the clinic.

Our prior study also showed that E-stim applied on the medial malleolus, targeting the calcaneal branch of the distal-tibial nerve, enhanced the lateral standing balance (Azbell et al., 2019, 2020). We expect that it is because E-stim, augmenting plantar cutaneous feedback on the heel, improved plantar sensitivity to the body sway (Machado et al., 2017). In our prior study, we also investigated the effect of a dual-task cognitive distraction, which was proved as an important factor in securing the effect of E-stim on the medial malleolus. However, we have not investigated the optimal way of applying the E-stim yet. In our prior study, plantar cutaneous augmentation was applied in a discrete mode (i.e., on/off) based on a predefined threshold, mainly for simplicity in the proof-of-concept study (Azbell et al., 2020). However, this discrete type of E-stim compromises its sensitivity on the lateral sway for simplicity in its implementation, and it is important to investigate if there is a better way of applying E-stim to improve lateral balance.

In this study, we investigated the best form of electrotactile feedback in augmenting plantar cutaneous feedback and improving lateral standing balance in challenging environments. We compared the efficacy between the discrete-mode and the proportional-mode mapping functions in applying the sway-based electrical stimulations. For the discrete-mode mapping, we turned on or off the E-stim based on the predefined threshold of the lateral board sway (see the left-side graph of Figure 1), while the E-stim was applied as proportional to the lateral board sway for the proportional-mode mapping (Zhao et al., 2020) (see the right-side graph of Figure 1). Our first hypothesis is that electrotactile feedback evoked on plantar area, indicating the lateral board sway, will promote lateral standing balance under dual-task cognitive distraction. Note that plantar cutaneous feedback is intrinsically associated with the balance function, and effectively enhances balance with cognitive distraction, as demonstrated in our prior study (Azbell et al., 2020). Our second hypothesis is that the proportional-mode electrotactile feedback will be more effective in improving lateral standing balance than its discrete-mode (i.e., on/off) counterpart. This is because electrotactile feedback on the foot would compensate for the somatosensory feedback from the lower limb better when it is proportionally changing like natural somatosensory feedback (D’Anna et al., 2019).

**Figure 3:**
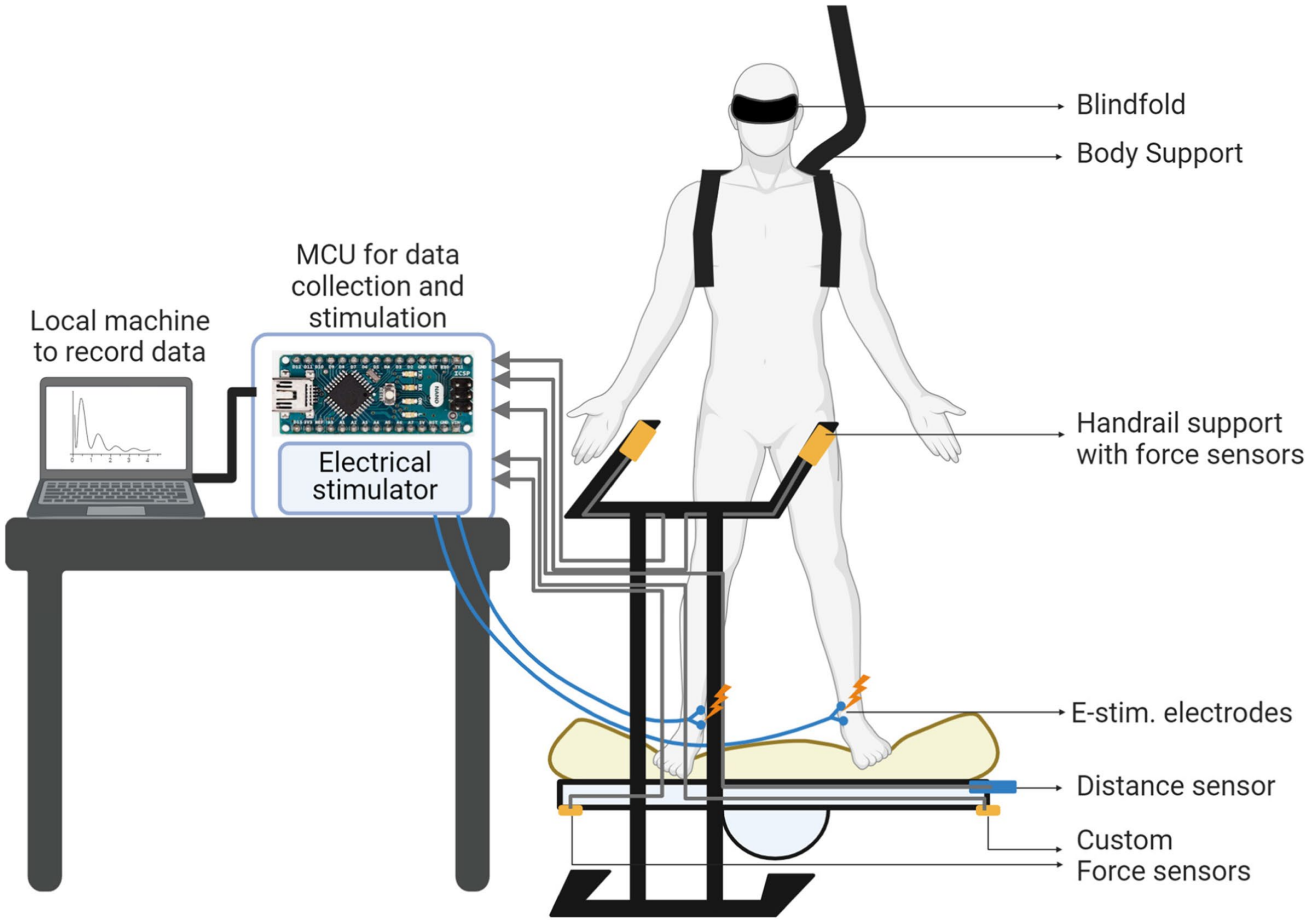
Overall experimental setup for the balancing test with balance board and desktop electronics.

Note that we designed the test condition with a balance board, which can provide a challenge to keep lateral balance (see Figure 3). As the study participants were healthy individuals without known balance issues, it was critical to provide the challenging balance condition to test the effect of sensory augmentation. We also designed the condition of compromised sensory feedback, with a blindfold and a foam pad on a balance board, which would provide a further challenge on balance. Multiple prior studies already demonstrated that plantar cutaneous feedback plays an important role in challenging balance conditions, while its role was underrepresented in normal balance conditions for healthy subjects (Corriveau et al., 2000; Bolton and Misiaszek, 2009; Park et al., 2019).

## 2 System implementation

### 2.1 Customized balance board

To measure the lateral standing balance under challenging ground conditions, we used a lateral balance board (3B Scientific W15075 Eucalyptus Wood Lateral Balance Rocker Board). A 4-inch foam padding was integrated on top of the balance board to reduce the plantar cutaneous feedback useful for balancing. Two custom-made force sensors were integrated on either side of the board to detect when the board touched the ground. A distance sensor was integrated on the left edge of the balance board to monitor the distance between each side of the board and the ground (i.e., lateral board sway). We also installed custom-made force sensors on both sides of the handrail to detect the timing when the subject’s hand left the handrails. A safety handrail was installed in front of the balance board for subjects to maintain balance before the start signal.

### 2.2 Desktop electronics

Two Arduino Nano microcontroller units (MCUs) and a computer have been employed and placed on the table near to the balance board. They performed all the necessary computations for data collection and stimulation. The first MCU was implemented as a state machine collecting the sensor data and determining the stimulation frequency based on the sensor data. The second MCU generated the control signal of the stimulator. The computer system interacted with the subject through the sensors and implemented the closed-loop operation, monitoring the board-to-ground distance and providing electrotactile feedback with a frequency inversely proportional to the board-to-ground distance (i.e., lateral board sway). In Figure 3, we show the overall experimental setup with the balance board and the electronics.

## 3 Experimental design

Ten healthy human subjects (nine male subjects and one female subject) participated in this study following the procedure described in the protocol approved by the Texas A&M University Institutional Review Board (IRB2018-1511F) on March 19th, 2019. The ages of all 10 subjects ranged from 23 to 26 years, with an average age of 24.7 years. All subjects were prescreened with the following exclusion criteria: use of any electronic implantable medical device, being sensitive or allergic to any kind of skin adhesive, having history of neurological disease or disorder, being sensitive to motion induced sickness, having problem in standing or walking, having any diagnosed cognitive impairment, being pregnant, and being a prisoner.

### 3.1 Experiment 1: identifying the site of stimulation

The first task was determining the proper site of stimulation on the subject’s feet. We picked the locations along the medial-calcaneal nerve that innervates onto the side of the heel (region A in Figure 2) and the posterior side of the heel (region B in Figure 2). We placed two electrodes along the medial-calcaneal nerve, one at inferior and posterior to the medial malleolus (i.e., branching point from the distal-tibial nerve) and the other at ~38 mm below the first one (i.e., branching point from the medial-calcaneal nerve), as shown in Figure 2 (Torres and Ferreira, 2012; Tang and Bordoni, 2021). Note that skin region around the medial-calcaneal nerve provides spatial selectiveness while avoiding the callus area with high contact impedance. Each electrode location was then slightly adjusted per subject to clearly evoke the electrotactile feedback on the region A or B (area innervated by the branches of the medial-calcaneal nerve). The stimulation used for identifying the site of stimulation was a bi-phasic voltage stimulus with a 1-ms pulse-width at 100-Hz frequency passing through a pair of electrodes, based on the prior success on augmenting plantar cutaneous feedback (Azbell et al., 2020).

### 3.2 Experiment 2: identifying the parameters of stimulation

Once the site of stimulation and the location of electrodes have been determined, the parameters of the E-stim were decided. The amplitude of E-stim was decided using the voltage level. The frequency was set to 100 Hz based on prior successes in sensory augmentation (Azbell et al., 2020; Zhao et al., 2020). The voltage level was slowly increased from zero while subjects were asked to provide verbal confirmation when they first felt the evoked sensation (*V_min_*) as well as when the E-stim became uncomfortable (*V_max_*). The two-thirds point of this range is set as the voltage for the experiment, as in Eq. 1, to evoke sensation without discomfort. Also, this setting secures the electrotactile perception to be between the notice and discomfort over 10–100 Hz, considering the small change in perception and discomfort thresholds over 10–100 Hz (Manoharan and Park, 2024).


(1) $$V_{\text{experiment}}=V_{\text{min}}+\frac{2}{3}*\left(V_{\text{max}}-V_{\text{min}}\right)$$

We also decided the frequency range that subjects can differentiate easily and can be used for the proportional E-stim. First, we set the minimum frequency as 10 Hz (*F_min_*) because any frequency lower than 10 Hz can be hardly used for the sub-second level decision in balancing task. We then slowly increased the frequency by 10 Hz steps, until the subject was unable to differentiate between the two neighboring steps in the frequency range. We set this maximal differentiable frequency (with 10 Hz step) as the maximum frequency (*F_max_*). This range of frequency from *F_min_* to *F_max_* was mapped to the balance board sway in such a way that the frequency of stimulation for each foot increases as each side of the board approaches to the ground. In case of discrete stimulation, the frequency was set to 100 Hz when the board-to-ground distance was outside of the balanced region (i.e., the board was swayed to one side) and no E-stim was applied when the board-to-ground distance was within the balanced region (i.e., the board was level).

### 3.3 Experiment 3: the balancing task

Subjects were blindfolded and asked to stand on the foam-clad balance board. They were also asked to hold onto the handrail in front of them and maintain the balance board at level. They were then asked to release the handrail and balance for as long as possible. At the same time, they were asked to perform the n-back counting task (counting backward by 7 from a random number) to interfere with cognitive engagement during the balancing task (Azbell et al., 2020). Each attempt at balancing was considered as a trial. There was a total of 150 trials with a 5-s break between each trial and a 5-min break between every 50 trials, to minimize the effect of fatigue.

**Figure 4:**
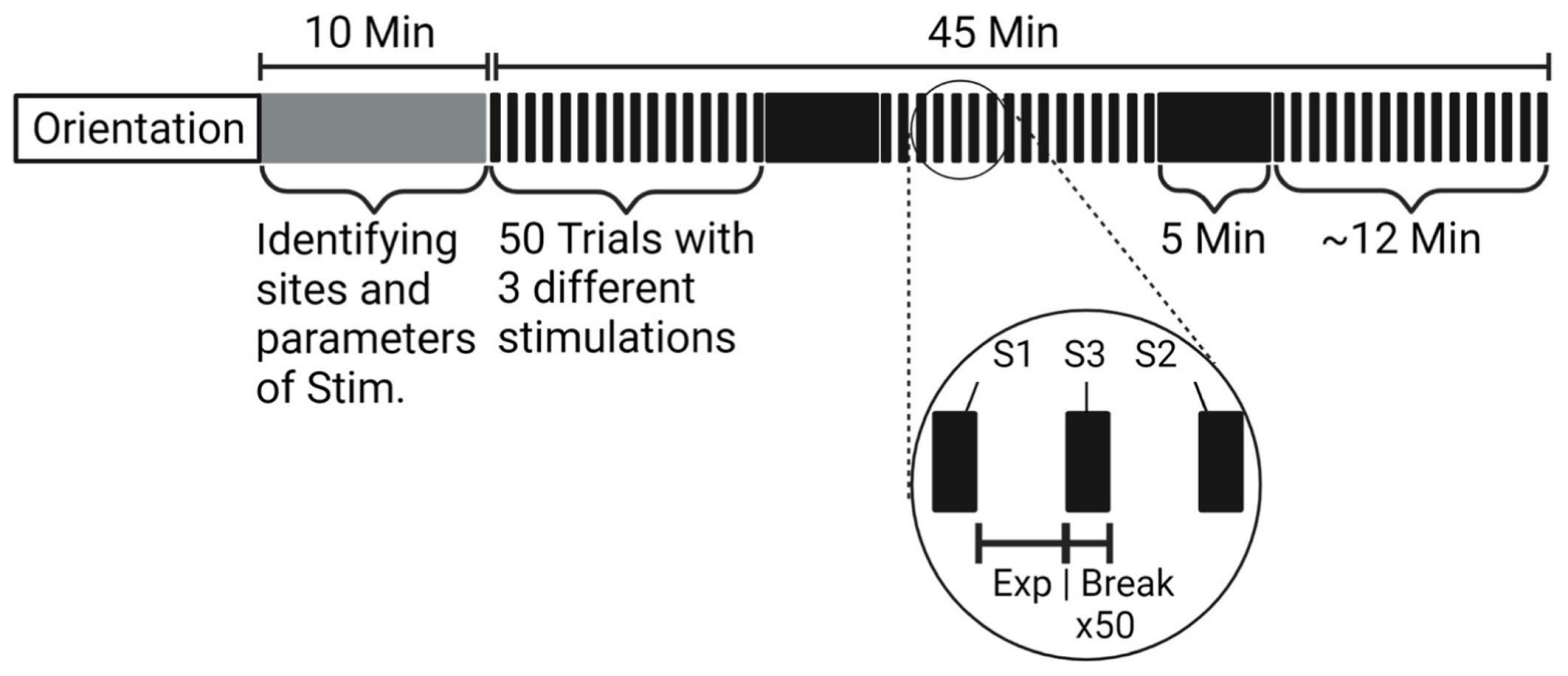
Detailed procedure and timeline of the experiment.

Trials using a balance board were conducted with three types of intervention. The 1st type was no stimulation, the 2nd type was discrete-mode stimulation, and the 3rd type was proportional-mode stimulation. E-stim was applied to the swayed side of the foot to assist the original somatosensory feedback from the feet and legs. The stimulation type for each trial was selected in random order but the three types of stimulation were distributed evenly and equally over the 150 trials (see Figure 4).

The discrete-mode stimulation was given as a constant 100-Hz E-stim, when the amount of sway exceeds the pre-defined threshold. That is, E-stim was activated when the board-to-ground distance was outside of the balanced region with more than 5-mm offset (i.e., balance board sway exceeds the ±5-mm threshold). The proportional-mode stimulation was given with the frequency of stimulation inversely proportional to the board-to-ground distance. The frequency of E-stim increased when each of the sides of the board approached the ground, and decreased when the board was closer to being level. As subjects perceived a low-frequency E-stim as a pulsing sensation and were able to differentiate the frequency difference (Azbell et al., 2020; Zhao et al., 2020), frequency modulation was used to deliver the analog information such as the lateral board sway. This method mimics the natural sensation of the plantar pressure when the body sways from one side to the other. A detailed timeline of the experiment is described in Figure 4. Note that subjects were not informed about the type of stimulation applied to their feet for each trial.

**Figure 5:**
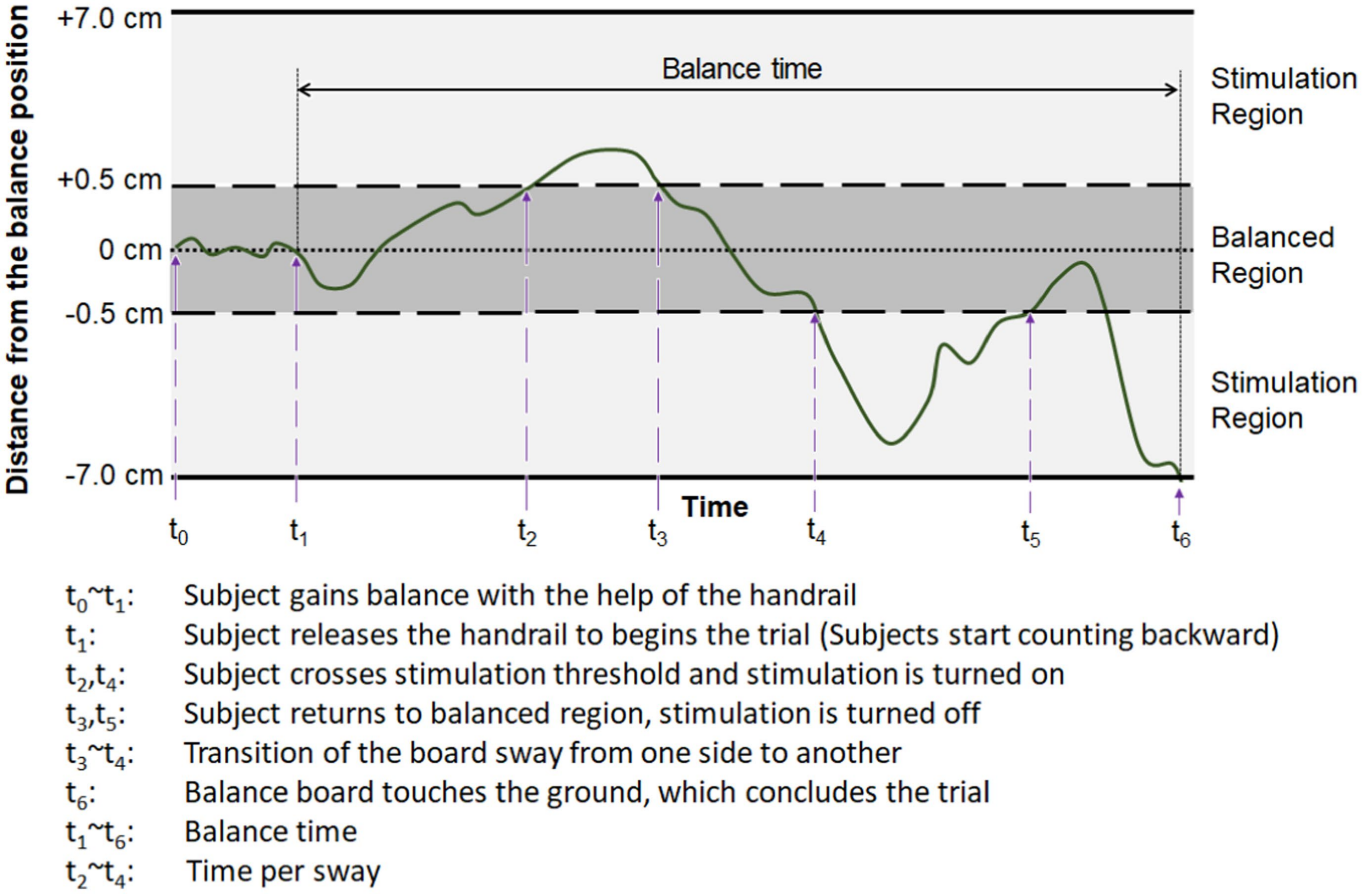
Exemplary change of the measured distance from the balance position (level), with definitions of output parameters used to evaluate the subject’s balancing capability on the balance board.

The effect of the E-stim on enhancing the lateral standing balance was evaluated by the subject’s balancing capability on the balance board, with balance time and time per sway as described in Figure 5. “Balance time (s)” is a basic measure, as the longer balance time suggests improvement in balancing capability. To further evaluate the balance during the balance time, we also measured “Time per sway (s).” This measure indicates the time duration that subjects maintained the sway direction before being changed to the other direction (e.g., left to right). Note that the change of sway direction was detected when the amount of sway exceeded the thresholds (see Figure 5). Although this new measure cannot still provide how each body part responded to the sensory feedback, it provides important information on how each subject maintained their balance before the balance board hit the ground.

### 3.4 Statistical analysis

To determine the efficacy of the independent factors on dependent variables, and to account for both within-subject and across-subject variability, we performed a linear mixed model analysis (SPSS, IBM, Chicago, IL, USA) per each experimental result. Both subjects and trials were set as random factors. For the independent factor (stimulation type), its effect on the dependent variables (balance time or time per sway) was determined. Kolmogorov–Smirnov test was used to check the normality of datasets, which satisfied the condition of *p* > 0.05. The significance level was set at 0.05 (95% confidence interval). All statistical data are reported as Mean ± STE (standard error) in the experimental result section and all statistical comparison results are shown with the corresponding *p* values.

## 4 Experimental results

### 4.1 E-stim on the medial-calcaneal nerve evoked electrotactile feedback at region A on the medial side of the heel

**Figure 6:**
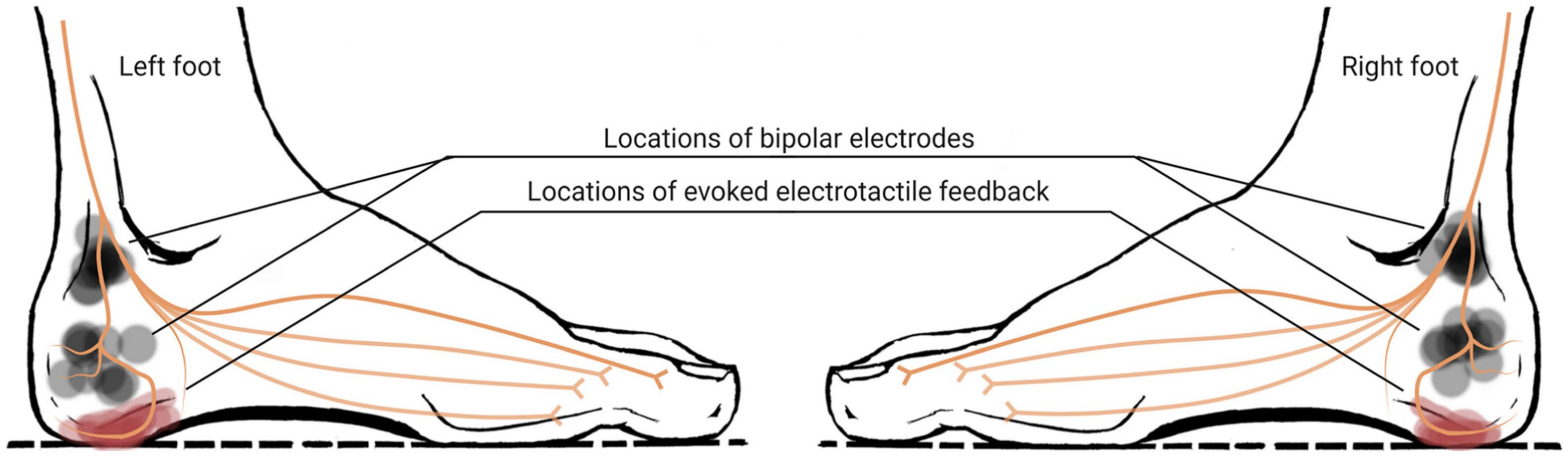
Actual locations of electrodes on each foot (semi-transparent grey circle indicates the electrode location for each subject) and evoked electrotactile feedback on each foot reported by each of 10 subjects, overlapped with a general nerve innervation diagram.

Our goal was to have the same region and strength of E-stim in both feet of each subject. We also selected the region for electrotactile feedback to be close to the heel. E-stim applied between those electrode locations successfully evoked electrotactile feedback in region A for all 10 subjects, and therefore we decided to select the region A as a region for electrotactile perception. Note that, four of 10 subjects reported light electrotactile feedback in the region B together with that in the region A (see Figure 3). A visual representation of the actual locations of electrodes selected for each subject, along with the locations of perception, is depicted in Figure 6.

### 4.2 Frequency range for proportional-mode stimulation was selected as 10–70 Hz

The voltage levels of E-stim for the 10 subjects were in the range of 11.7 V and 23.5 V with an average of 18.5 V, median of 18 V, and standard deviation of 3.24 V. All 10 subjects were able to differentiate the E-stim until 70 Hz and failed to distinguish between 70 Hz and 80 Hz. Therefore, the frequency range for proportional-mode stimulation was selected as 10–70 Hz.

### 4.3 Proportional-mode E-stim increased balance time while discrete-mode E-stim did not

**Figure 7:**
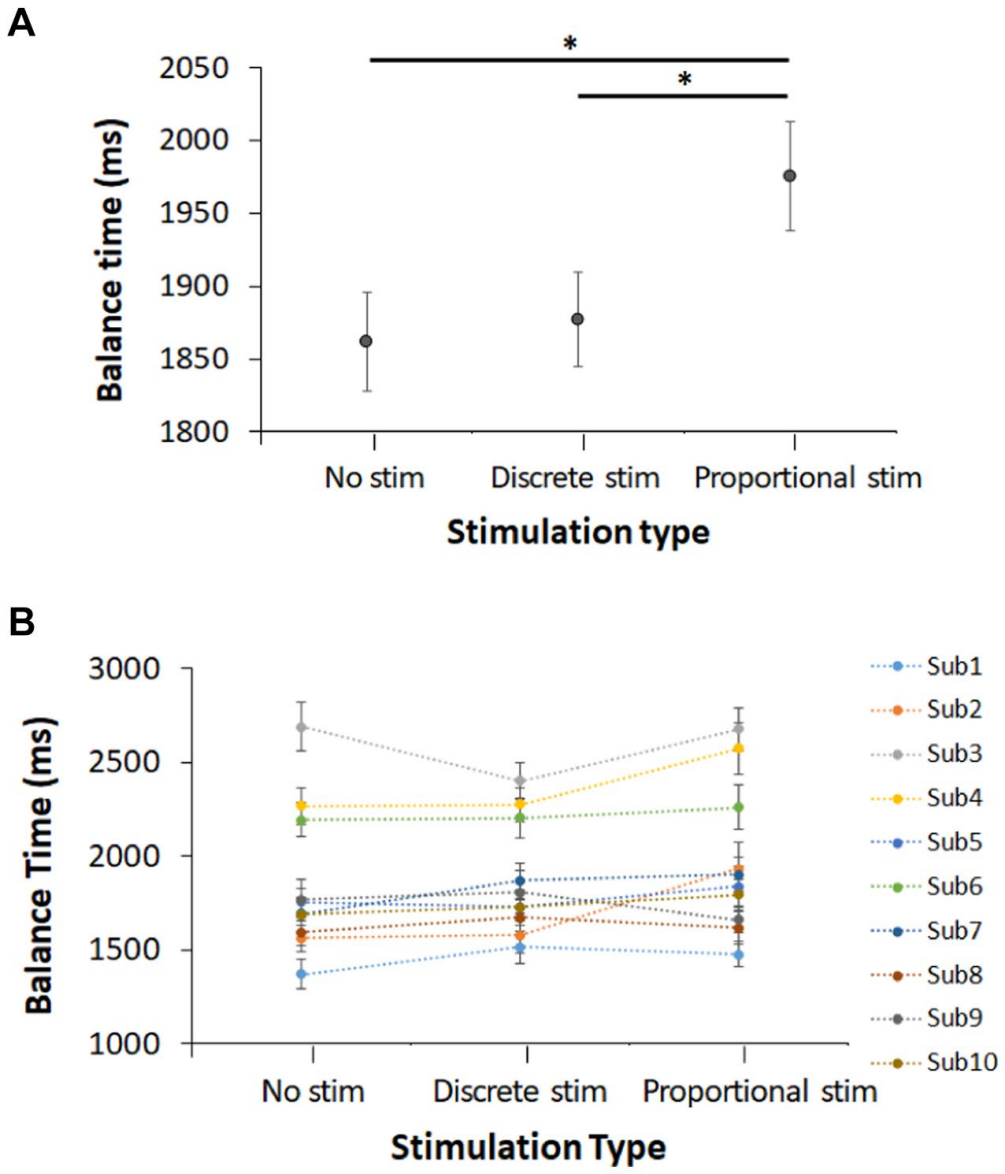
Balance time of 10 subjects for each of the three stimulation conditions (no stimulation, discrete-mode stimulation, and proportional-mode stimulation): **(A)** overall balance time and **(B)** individual balance time (all values are represented as Mean ± STE). * indicates that difference between these two means is statistically significant with 95% confidence interval.

The experiment was conducted with 10 healthy human subjects and the average with standard error across all subjects and their data are shown in Figures 7A,B, respectively. We observed that the control trials (no stimulation) resulted in a balance time of 1.86 ± 0.03 s. Discrete-mode stimulation results in a balance time of 1.88 ± 0.03 s and proportional-mode stimulation results in a balance time of 1.98 ± 0.04 s. Proportional-mode stimulation increased balance time by 6.5% from baseline. Statistical test suggests that balance time with the proportional electrotactile feedback is longer than that with no stimulation (*p* = 0.010). The balance time with the proportional electrotactile feedback is also longer than that with its discrete counterpart (*p* = 0.037). However, the balance time with the discrete electrotactile feedback was not statistically different from that without any stimulation (*p* = 0.669).

### 4.4 Proportional-mode E-stim increased time-per-sway while discrete-mode E-stim did not

**Figure 8:**
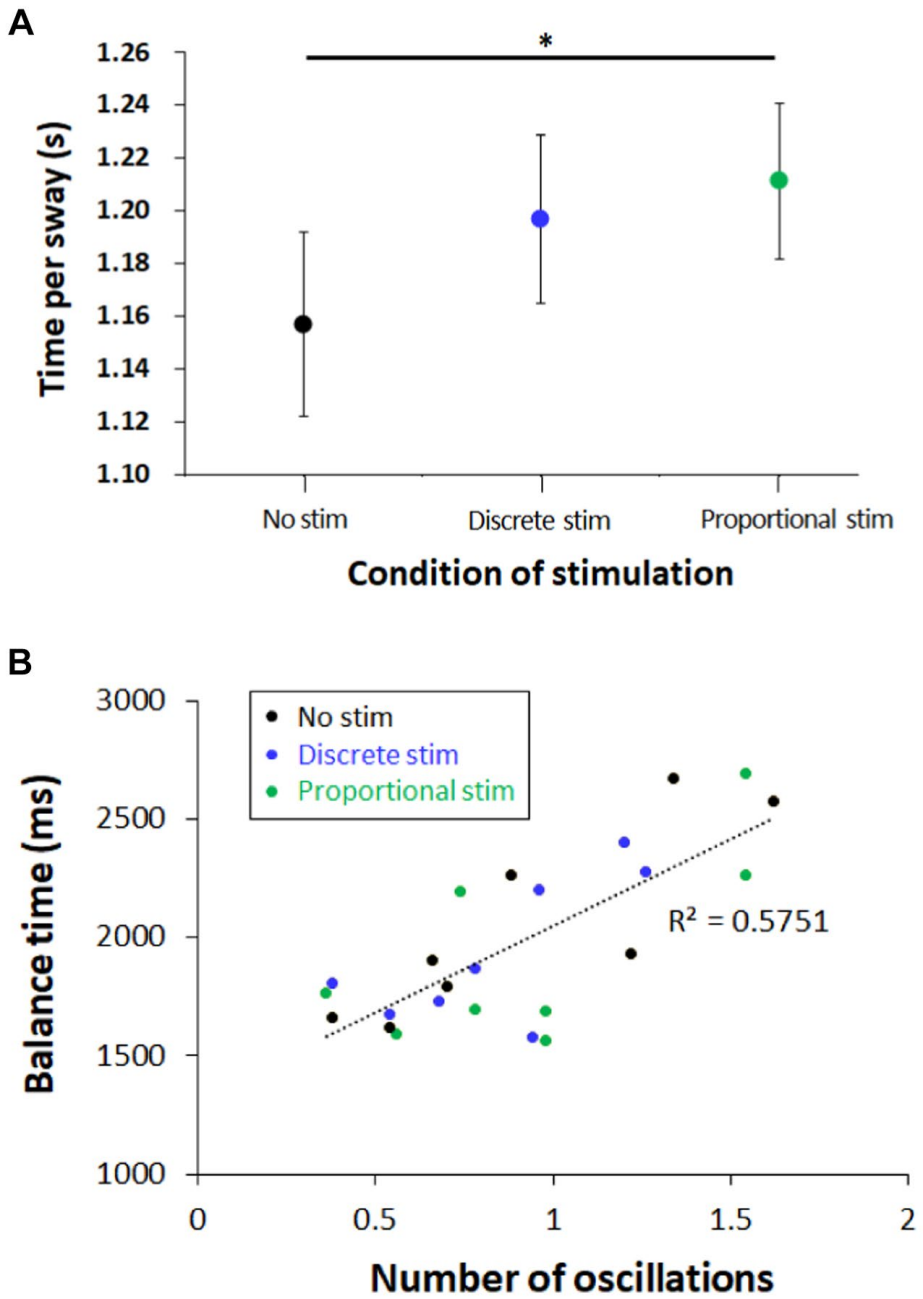
Graphs for **(A)** time per sway per each condition of stimulation and **(B)** Relationship between the number of oscillation and the balance time. Each data point indicates different subject with color code indicating the condition of stimulation. The “oscillation” indicates the oscillation of the balance board swaying from one side to the other side. The board is “swaying” to one side when the measured distance at the sensor exceeds the predefined threshold of ±5 mm. Note that 8 of 10 subjects’ data were available for this analysis. * indicates that difference between these two means is statistically significant with 95% confidence interval.

Time per sway was measured as 0.157 ± 0.035 (s), 0.197 ± 0.032 (s), and 0.211 ± 0.029 (s), for conditions of no stimulation, discrete-mode stimulation, and proportional-mode stimulation, respectively (see Figure 8A). Time per sway was increased by proportional-mode stimulation compared to the baseline measure without stimulation (*p* = 0.035), while it was not by discrete-mode stimulation (*p* = 0.053).

### 4.5 Strong positive correlation between the number of oscillations and the balance time

We analyzed data to calculate how many times the balance board oscillated between each side during each trial. The average number of oscillations was 0.90 ± 0.50 (MEAN ± STE), which suggests that the balance board oscillated only once during ~2 s of balance time. We also depicted the relationship between the number of oscillations and the balance time at Figure 8B, using the data points per each subject per each condition. Despite the small number of oscillations, we observed a strong positive correlation between the number of oscillations and the balance time.

### 4.6 Comparison with prior works that employed visual/auditory augmentation

It would be important to compare the effectiveness of the proposed E-stim with prior sensory augmentation approaches represented by visual/auditory augmentation (Zijlstra et al., 2010). As the balance board setup is unique for this work, it is hard to compare the results directly with prior works. Therefore, we indirectly compared the results with related dynamic stability measures. To evaluate dynamic stability, prior works with visual/auditory augmentation employed the movement amplitude of the center of pressure (CoP) and time per sway. Although the presented work does not present the CoP movement, the balance time is closely related to the CoP movement and represents dynamic stability. Table 1 shows the comparison between the presented and prior works that reported the effect of visual/auditory augmentation on standing balance, as a measure of the movement amplitude of CoP and the time per sway (Lajoie, 2004; Santarmou et al., 2006; Hatzitaki et al., 2009; Vos et al., 2022). These indirect comparison results suggest that the presented electrotactile augmentation approach showed effectiveness comparable to those of visual/auditory augmentation.

**Table 1:** Comparison table between the presented work and prior works regarding the effect of sensory augmentation (visual, auditory, or electrotactile augmentation) on dynamic balance measures at standing posture.

	Vos et al. (2022) (visual)	Hatzitaki et al. (2009) (visual)	Lajoie (2004) (visual)	Santarmou et al. (2006) (auditory)	This work (electrotactile)
Δ Balance time	–	–	–	–	6.5%
Δ CoP amplitude	6%	16%	–	13%	–
Δ Time per sway	–	–	4.2%	–	4.8%

All values are the amount of improvement of the experimental group as a ratio compared to the values of the control group.

## 5 Discussion

### 5.1 Proportional-mode E-stim enhanced the lateral standing balance at challenging balance condition

The experimental results in this study support the first hypothesis that electrotactile feedback evoked on the plantar area, indicating the lateral board sway, promotes lateral standing balance at dual-task cognitive distraction. The balance time was increased when the E-stim was applied with a frequency proportional to the lateral board sway. This result suggests that proportional-mode E-stim was useful in maintaining standing balance at challenging balance conditions. Further, the time duration for each state was also increased with proportional-mode E-stim. This result suggests that subjects maintained the balance for a longer time duration while being swayed to one side. These results confirm the importance of plantar cutaneous feedback on lateral balance, which has been often underrepresented perhaps because of the improper experimental setting to test its importance (Höhne et al., 2009, 2011). Note that plantar cutaneous feedback plays an important role in balancing under challenging locomotor conditions, as shown in multiple animal studies (Bouyer and Rossignol, 2003; Bolton and Misiaszek, 2009). Balancing tasks on the balance board are challenging even for healthy subjects, as the board is in a metastable state and a tiny amount of lateral asymmetry would sway the board to one side. In the challenging standing conditions for balancing, as well as challenging locomotor conditions, plantar cutaneous augmentation using E-stim seems effective in a consistent manner (Azbell et al., 2019, 2020).

### 5.2 Proportional-mode E-stim was more effective than discrete-mode E-stim on improving standing balance

As shown in Figure 7, the balance time of subjects with proportional-mode E-stim was longer than that with discrete-mode E-stim, which supports our second hypothesis. The time-per-state data in Figure 8A also support the second hypothesis, as proportional-mode E-stim increased the time duration per each swayed state while discrete-mode E-stim did not. It is perhaps because the proportional-mode E-stim better mimics the natural somatosensory feedback responding to the board sway than its discrete counterpart. Note that proprioceptive feedback, which plays a critical role in balancing, changes continuously rather than discretely. Pressure feedback at the plantar surface also changes continuously rather than discretely. As E-stim was used to provide useful information for balance, its proportional-mode application would be better suited to assist the natural somatosensory feedback than the discrete-mode application.

### 5.3 Subjects having a greater number of oscillations between the two sides stayed longer on the balance board

The strong positive correlation between the number of oscillations and the balance time indicates that subjects having more oscillation between the two sides stayed longer on the balance board. As subjects will have a higher chance to oscillate between the two sides with a longer balance time, this result seems natural. However, the average number of oscillations tripled (0.5–1.5) while balance time was increased only by ~50%. In other words, the average number of oscillations increased much more steeply than the increase in balance time. This result suggests that the number of oscillations plays an important role in maintaining the lateral standing balance on the balance board. However, it is hard to associate the number of oscillations with the effect of stimulation, as cases with and without stimulation showed similar tendencies in between (see Figure 8B). Considering the time duration per each swayed state increased by the application of proportional-mode E-stim (see Figure 8), electrotactile augmentation seems more effective on maintaining the balance at the swayed state rather than increasing the capability of dynamic oscillation.

### 5.4 Medial-calcaneal nerve stimulation provides consistent electrotactile feedback on the medial side of the heel

Medial-calcaneal nerve stimulation seems like a good choice to evoke electrotactile feedback on the heel, not just because it is consistent over the plantar pressure but also because it is consistent over the subjects. All 10 subjects reported that they felt sensations evoked in the region A, for both feet, by the stimulation applied between the electrodes located along the medial-calcaneal nerve (see Figures 3, 6). As electrodes were easily placed using the anatomical landmarks: one inferior and posterior to the medial malleolus and the other at ~38 mm below the first one, we expect that the electrotactile feedback in region A would be consistently evoked for the public. Note that four of 10 subjects reported a slight sensation in region B on their right foot, on top of the sensation in region A. However, considering the anatomical variations of the foot and its varying nerve innervation, the consistent electrotactile feedback in region A is still remarkable. Importantly, the E-stim in region A evokes not just electrotactile feedback but frequency-dependent electrotactile feedback, which can be used to provide continuous data proportional to the sway.

### 5.5 Limitation and future direction

#### 5.5.1 Optimal location of electrotactile feedback for balance should be further investigated

We initially thought to augment the sensation on the heel of the foot, by stimulating the inferior calcaneal nerve (see Figure 3) as in our prior work (Azbell et al., 2020). This is because the tactile feedback on the heel is directly associated with the balancing function. However, subjects reported that the electrotactile sensation was significantly reduced when pressure was simultaneously applied to the heel. To avoid this sensory reduction, we augmented the sensation on the medial side of the heel not contacting the ground, by stimulating the medial-calcaneal nerve (see Figures 3, 6). However, we do not exclude the possibility that the reduced electrotactile feedback by plantar pressure may still be effective in improving balancing. As our prior work stimulating inferior calcaneal nerve (Azbell et al., 2020) showed balance improvement even with discrete electrotactile feedback, this certainly needs further investigation.

#### 5.5.2 Parameters of E-stim should be carefully examined

It should be noted that the discrete-mode E-stim showed a statistically meaningful increase in balance time in our prior study (Azbell et al., 2020), which does not agree with the result in this study. This discrepancy can be explained in two ways: First, it may be due to the difference in the threshold used to start the E-stim. A 5-mm threshold (5-mm change from the level) was used in this work while we used a 15-mm threshold for our prior study (Azbell et al., 2020). Perhaps the threshold is critical for the efficacy of discrete-mode E-stim. Second, the region of stimulation used in this experiment was different from the region used in our prior study (Azbell et al., 2020). While our prior study evoked sensation on the heel contacting the ground, the E-stim used in this study evoked sensation on the medial side of the heel not contacting the ground (see region A in Figure 3). To achieve the consistent effect of E-stim on balance improvement, parameters for applying E-stim, such as location, amplitude, and frequency, should be carefully examined in the future study.

#### 5.5.3 Training with E-stim may benefit people with peripheral neuropathy and people experiencing uncontrollable body sway

The presented E-stim approach can potentially benefit people with peripheral neuropathy, as they often suffer from sensory deficits and balance problems. As patients suffering from peripheral neuropathy (e.g., diabetic neuropathy) have limited sensory capability at the distal part of their limbs, the progression of the peripheral neuropathy should be carefully examined before applying the presented E-stim approach. Although the calcaneal nerve is a better location than the feet as the sensory disturbance starts in the distal segment (feet), even the calcaneal nerve can be affected by the progression of the peripheral neuropathy, which will then negate the effectiveness of the presented E-stim approach.

Also, the presented training with E-stim would help people to keep balance in challenging conditions. Note that people experience unexpected body sway in daily environments, by uneven ground conditions and sudden acceleration/deceleration of public transportation. If they have insufficient muscular power or compromised sensory feedback, they hardly control body sway and end up with falls. The training with E-stim presented in this study can be applied to these people to avoid undesirable falls and hospitalization.

#### 5.5.4 Other limitations and future directions

First, this study is limited with the number of subjects (*N* = 10), although this number still provided statistical significance to interpret the effect of the proportional-mode E-stim on the lateral balance. Therefore, a more detailed between-subject analysis with a larger number of subjects is necessary to confirm the result. The gender effect should be investigated too with balanced recruitment of male and female subjects. Second, the participants of the study were limited to healthy young individuals. Follow-up studies are necessary to determine if the findings can be applied to populations with sensory impairment. Third, this study did not measure aftereffects of the training. The performance improvement can be retained after the training (i.e., training aftereffects), and further research is required to clarify the aftereffects. Fourth, we did not evaluate the importance of balance time at the presented balancing board task. For example, the increase by proportional-mode E-stim (~150 ms) can be critical in balancing time considering that the falling event occurs based on the reaction in sub-second duration. Therefore, future experiments should be designed to test the balancing function in a multifaceted way. Fifth, we did not assess the percentage of correct answers during the n-back counting task. As the level of distraction would affect the performance, it would be interesting to see if there is any correlation between the percentage of correct answers and the balancing capability. Sixth, the current method of testing the frequency discriminability is limited with potential subjective influence and limited precision (10 Hz step). In future experiments, we will employ two-alternative forced choice (2AFC) or just-noticeable difference (JND) to better test the frequency discriminability.

Despite all the above limitations of this study, we observed that the transcutaneous medial-calcaneal nerve stimulation successfully augmented plantar cutaneous feedback and improved balance. The E-stim was applied via the skin near the medial malleolus, in a fully non-invasive and minimally intrusive way. Also, we found that proportional electrotactile feedback, changing its frequency according to the amount of sway, better improves the lateral standing balance than its discrete counterpart. We expect that this new method of plantar cutaneous augmentation will be easily added to the list of existing clinical therapies, once we demonstrate its efficacy on people with diabetes or stroke, who have critical issues in the balance.

## Data availability statement

The raw data supporting the conclusions of this article will be made available by the authors, without undue reservation.

## Ethics statement

The studies involving humans were approved by Texas A&M University Institutional Review Board. The studies were conducted in accordance with the local legislation and institutional requirements. The participants provided their written informed consent to participate in this study.

## Author contributions

HP, S-HC, and VR conceptualized the idea and designed the experiment. VR prepared an electrical system for experiments and recruited human subjects and collected experimental data. VR and HP wrote the experimental protocol approved for human subject experiments. VR, JK, S-HC, YC, and HP analyzed experimental data. RK, JK, S-HC, YC, and HP wrote the manuscript. All authors contributed to the article and approved the submitted version.

## References

[ref1] Andersson G. Hagman J. Talianzadeh R. Svedberg A. Larsen H. C. (2002). Effect of cognitive load on postural control. Brain Res. Bull. 58, 135–139. doi: 10.1016/S0361-9230(02)00770-0 12121823

[ref2] Angeli C. A. Boakye M. Morton R. A. Vogt J. Benton K. Chen Y.. (2018). Recovery of over-ground walking after chronic motor complete spinal cord injury. N. Engl. J. Med. 379, 1244–1250. doi: 10.1056/NEJMoa1803588, PMID: 30247091

[ref3] Azbell J. Park J. K. Chang S. H. Engelen M. P. Park H. Closed-loop tactile augmentation by transcutaneous stimulation on either the foot sole or the palm to improve lateral postural balance. 9th international IEEE/EMBS conference on neural engineering (NER) (2019) San Francisco, USA

[ref4] Azbell J. Park J. Chang S. H. Engelen M. P. Park H. (2020). Plantar or palmar tactile augmentation improves lateral postural balance with significant influence from cognitive load. IEEE Trans. Neural Syst. Rehabil. Eng. 29, 113–122. doi: 10.1109/TNSRE.2020.3037128 33170781

[ref5] Bae K. H. Shin J. H. Lee J. S. Yang J. O. Lee B. J. Park S. B. (2016). Analyses of plantar foot pressure and static balance according to the type of insole in the elderly. Korean J. Sport Biomech. 26, 115–126. doi: 10.5103/KJSB.2016.26.1.115

[ref6] Ballardini G. Florio V. Canessa A. Carlini G. Morasso P. Casadio M. (2020). Vibrotactile feedback for improving standing balance. Front. Bioeng. Biotechnol. 8:94. doi: 10.3389/fbioe.2020.00094, PMID: 32154229 PMC7046798

[ref7] Bernard-Demanze L. Vuillerme N. Ferry M. Berger L. (2009). Can tactile plantar stimulation improve postural control of persons with superficial plantar sensory deficit? Aging Clin. Exp. Res. 21, 62–68. doi: 10.1007/BF03324900, PMID: 19225271

[ref8] Bolton D. A. Misiaszek J. E. (2009). Contribution of hindpaw cutaneous inputs to the control of lateral stability during walking in the cat. J. Neurophysiol. 102, 1711–1724. doi: 10.1152/jn.00445.2009, PMID: 19605609

[ref9] Bouyer L. J. Rossignol S. (2003). Contribution of cutaneous inputs from the hindpaw to the control of locomotion. II. Spinal cats. J. Neurophysiol. 90, 3625–3639. doi: 10.1152/jn.00496.2003, PMID: 12944536

[ref10] Charkhkar H. Christie B. P. Triolo R. J. (2020). Sensory neuroprosthesis improves postural stability during sensory organization test in lower-limb amputees. Sci. Rep. 10:6984. doi: 10.1038/s41598-020-63936-2, PMID: 32332861 PMC7181811

[ref11] Chen G. Chan C. K. Guo Z. Yu H. (2013). A review of lower extremity assistive robotic exoskeletons in rehabilitation therapy. Crit. Rev. Biomed. Eng. 41, 343–363. doi: 10.1615/critrevbiomedeng.2014010453 24941413

[ref12] Corriveau H. Prince F. Hebert R. Raiche M. Tessier D. Maheux P.. (2000). Evaluation of postural stability in elderly with diabetic neuropathy. Diabetes Care 23, 1187–1191. doi: 10.2337/diacare.23.8.1187, PMID: 10937520

[ref13] D’Anna E. Valle G. Mazzoni A. Strauss I. Iberite F. Patton J.. (2019). A closed-loop hand prosthesis with simultaneous intraneural tactile and position feedback. Science. Sci. Robot. 4:eaau8892. doi: 10.1126/scirobotics.aau8892 33137741

[ref14] Dollahon D. Ryu S. C. Park H. A computational internal model to quantify the effect of sensorimotor augmentation on motor output. In 42nd annual international conference of the IEEE engineering in Medicine & Biology Society (EMBC) (2020), Canada 10.1109/EMBC44109.2020.9176109 33018817

[ref15] Ferris D. Sawicki G. Domingo A. (2005). Powered lower limb orthoses for gait rehabilitation. Top. Spinal Cord Inj. Rehabil. 11, 34–49. doi: 10.1310/6GL4-UM7X-519H-9JYD 16568153 PMC1414628

[ref16] Hatzitaki V. Amiridis I. G. Nikodelis T. Spiliopoulou S. (2009). Direction-induced effects of visually guided weight-shifting training on standing balance in the elderly. Gerontology 55, 145–152. doi: 10.1159/000142386, PMID: 18594127

[ref17] Höhne A. Stark C. Brüggemann G. P. (2009). Plantar pressure distribution in gait is not affected by targeted reduced plantar cutaneous sensation. Clin. Biomech. 24, 308–313. doi: 10.1016/j.clinbiomech.2009.01.001 19201070

[ref18] Höhne A. Stark C. Brüggemann G. P. Arampatzis A. (2011). Effects of reduced plantar cutaneous afferent feedback on locomotor adjustments in dynamic stability during perturbed walking. J. Biomech. 44, 2194–2200. doi: 10.1016/j.jbiomech.2011.06.012, PMID: 21726865

[ref19] Hornby T. G. Zemon D. H. Campbell D. (2005). Robotic-assisted, body-weight–supported treadmill training in individuals following motor incomplete spinal cord injury. Phys. Ther. 85, 52–66. doi: 10.1093/ptj/85.1.52, PMID: 15623362

[ref20] Huang H. Wolf S. L. He J. (2006). Recent developments in biofeedback for neuromotor rehabilitation. J. Neuroeng. Rehabil. 3, 1–2. doi: 10.1186/1743-0003-3-11 16790060 PMC1550406

[ref21] Kelleher K. J. Spence W. D. Solomonidis S. Apatsidis D. (2010). The effect of textured insoles on gait patterns of people with multiple sclerosis. Gait Posture 32, 67–71. doi: 10.1016/j.gaitpost.2010.03.008, PMID: 20400312

[ref22] Kim M. Y. Kim J. H. Lee J. U. Yoon N. M. Kim B. Kim J. (2012). The effects of functional electrical stimulation on balance of stroke patients in the standing posture. J. Phys. Ther. Sci. 24, 77–81. doi: 10.1589/jpts.24.77

[ref23] Kouzaki M. Masani K. (2008). Reduced postural sway during quiet standing by light touch is due to finger tactile feedback but not mechanical support. Exp. Brain Res. 188, 153–158. doi: 10.1007/s00221-008-1426-5 18506433

[ref24] Lajoie Y. (2004). Effect of computerized feedback postural training on posture and attentional demands in older adults. Aging Clin. Exp. Res. 16, 363–368. doi: 10.1007/BF03324565 15636461

[ref25] Lynskey J. V. Belanger A. Jung R. (2008). Activity-dependent plasticity in spinal cord injury. J. Rehabil. Res. Dev. 45, 229–240. doi: 10.1682/JRRD.2007.03.0047, PMID: 18566941 PMC2562625

[ref26] Machado Á. S. Silva C. B. Rocha E. S. Carpes F. P. (2017). Effects of plantar foot sensitivity manipulation on postural control of young adult and elderly. Rev. Bras. Reumatol. 57, 30–36. doi: 10.1016/j.rbr.2015.11.005, PMID: 28137400

[ref27] Manoharan S. Park H. (2024). Characterization of perception by transcutaneous electrical stimulation in terms of tingling intensity and temporal dynamics. Biomed. Eng. Lett. 14, 35–44. doi: 10.1007/s13534-023-00308-5, PMID: 38186955 PMC10770012

[ref28] Najafi B. Talal T. K. Grewal G. S. Menzies R. Armstrong D. G. Lavery L. A. (2017). Using plantar electrical stimulation to improve postural balance and plantar sensation among patients with diabetic peripheral neuropathy: a randomized double blinded study. J. Diabetes Sci. Technol. 11, 693–701. doi: 10.1177/1932296817695338, PMID: 28627217 PMC5588835

[ref29] Nanivadekar A. C. Bose R. Petersen B. A. Okorokova E. V. Sarma D. Farooqui J.. (2022). Spinal cord stimulation restores sensation, improves function, and reduces phantom limb pain after transtibial amputation. med Rxiv. doi: 10.1101/2022.09.15.22279956

[ref30] National Institute of Neurological Disorders and Stroke (2018). Peripheral Neuropathy Fact Sheet. Bethesda, Maryland, United States: NIH Publication.

[ref31] Park H. Latash E. M. Molkov Y. I. Klishko A. N. Frigon A. DeWeerth S. P.. (2019). Cutaneous sensory feedback from paw pads affects lateral balance control during split-belt locomotion in the cat. J. Exp. Biol. 222:jeb198648. doi: 10.1242/jeb.198648 31308054 PMC6679349

[ref32] Pearson K. G. (2008). Role of sensory feedback in the control of stance duration in walking cats. Brain Res. Rev. 57, 222–227. doi: 10.1016/j.brainresrev.2007.06.014, PMID: 17761295

[ref33] Roemmich R. T. Long A. W. Bastian A. J. (2016). Seeing the errors you feel enhances locomotor performance but not learning. Curr. Biol. 26, 2707–2716. doi: 10.1016/j.cub.2016.08.012, PMID: 27666970 PMC5081226

[ref34] Santarmou E. Dozza M. Lannocca M. Chiari L. Cappello A. (2006). Insole pressure sensor-based audio-biofeedback for balance improvement. Gait Posture 24, S30–S31. doi: 10.1016/j.gaitpost.2006.09.048

[ref35] Shon A. Brakel K. Hook M. Park H. (2021). Fully implantable plantar cutaneous augmentation system for rats using closed-loop electrical nerve stimulation. IEEE Trans. Biomed. Circuits Syst. 15, 326–338. doi: 10.1109/TBCAS.2021.3072894, PMID: 33861705

[ref36] Shull P. B. Damian D. D. (2015). Haptic wearables as sensory replacement, sensory augmentation and trainer–a review. J. Neuroeng. Rehabil. 12, 1–3. doi: 10.1186/s12984-015-0055-z 26188929 PMC4506766

[ref37] Sigrist R. Rauter G. Riener R. Wolf P. (2013). Augmented visual, auditory, haptic, and multimodal feedback in motor learning: a review. Psychon. Bull. Rev. 20, 21–53. doi: 10.3758/s13423-012-0333-8, PMID: 23132605

[ref38] Sorgini F. Caliò R. Carrozza M. C. Oddo C. M. (2018). Haptic-assistive technologies for audition and vision sensory disabilities. Disabil. Rehabil. Assist. Technol. 13, 394–421. doi: 10.1080/17483107.2017.1385100, PMID: 29017361

[ref39] Takeoka A. Vollenweider I. Courtine G. Arber S. (2014). Muscle spindle feedback directs locomotor recovery and circuit reorganization after spinal cord injury. Cell 159, 1626–1639. doi: 10.1016/j.cell.2014.11.019, PMID: 25525880

[ref40] Tang A. Bordoni B. Anatomy, bony pelvis and lower limb, foot nerves. Treasure Island (FL): StatPearls Publishing. (2021) 30725977

[ref41] Torres A. L. Ferreira M. C. (2012). Study of the anatomy of the tibial nerve and its branches in the distal medial leg. Acta ortopedica brasileira. 20, 157–164. doi: 10.1590/S1413-78522012000300005, PMID: 24453596 PMC3718430

[ref42] Viseux F. Lemaire A. Barbier F. Charpentier P. Leteneur S. Villeneuve P. (2019). How can the stimulation of plantar cutaneous receptors improve postural control? Review and clinical commentary. Neurophysiol. Clin. 49, 263–268. doi: 10.1016/j.neucli.2018.12.006, PMID: 30639034

[ref43] Vos L. A. Prins M. R. Kingma I. (2022). Training potential of visual feedback to improve dynamic postural stability. Gait Posture 92, 243–248. doi: 10.1016/j.gaitpost.2021.11.040, PMID: 34883424

[ref44] Wagner F. B. Mignardot J. B. Le Goff-Mignardot C. G. Demesmaeker R. Komi S. Capogrosso M.. (2018). Targeted neurotechnology restores walking in humans with spinal cord injury. Nature 563, 65–71. doi: 10.1038/s41586-018-0649-2, PMID: 30382197

[ref45] Zhao Z. Yeo M. Manoharan S. Ryu S. C. Park H. (2020). Electrically evoked proximity sensation can enhance fine finger control in telerobotic pinch. Sci. Rep. 10, 1–2. doi: 10.1038/s41598-019-56985-9 31932709 PMC6957695

[ref46] Zijlstra A. Mancini M. Chiari L. Zijlstra W. (2010). Biofeedback for training balance and mobility tasks in older populations: a systematic review. J. Neuroeng. Rehabil. 7, 1–5. doi: 10.1186/1743-0003-7-58 21143921 PMC3019192

